# Neck Circumference, along with Other Anthropometric Indices, Has an Independent and Additional Contribution in Predicting Fatty Liver Disease

**DOI:** 10.1371/journal.pone.0118071

**Published:** 2015-02-13

**Authors:** Bi-xia Huang, Ming-fan Zhu, Ting Wu, Jing-ya Zhou, Yan Liu, Xiao-lin Chen, Rui-fen Zhou, Li-jun Wang, Yu-ming Chen, Hui-lian Zhu

**Affiliations:** 1 Faculty of Nutrition, School of Public Health, Sun Yat-sen University, Guangzhou, Guangdong Province, The People’s Republic of China; 2 Health Examination Centre, First Affiliated Hospital of Sun Yat-sen University, Guangzhou, Guangdong Province, The People’s Republic of China; University of Verona, Ospedale Civile Maggiore, ITALY

## Abstract

**Background and Aim:**

Previous studies have indicated that neck circumference is a valuable predictor for obesity and metabolic syndrome, but little evidence is available for fatty liver disease. We examined the association of neck circumference with fatty liver disease and evaluated its predictive value in Chinese adults.

**Methods:**

This cross-sectional study comprised 4053 participants (1617 women and 2436 men, aged 20-88) recruited from the Health Examination Center in Guangzhou, China between May 2009 and April 2010. Anthropometric measurements were taken, abdominal ultrasonography was conducted and blood biochemical parameters were measured. Covariance, logistic regression and receiver operating characteristic curve analyses were employed.

**Results:**

The mean neck circumference was greater in subjects with fatty liver disease than those without the disease in both women and men after adjusting for age (P<0.001). Logistic regression analysis showed that the age-adjusted ORs (95% CI) of fatty liver disease for quartile 4 (vs. quartile 1) of neck circumference were 7.70 (4.95-11.99) for women and 12.42 (9.22-16.74) for men. After further adjusting for other anthropometric indices, both individually and combined, the corresponding ORs remained significant (all *P*-trends<0.05) but were attenuated to 1.94-2.53 for women and 1.45-2.08 for men. An additive interaction existed between neck circumference and the other anthropometric measures (all *P*<0.05). A high neck circumference value was associated with a much greater prevalence of fatty liver disease in participants with both high and normal BMI, waist circumference and waist-to-hip ratio values.

**Conclusions:**

Neck circumference was an independent predictor for fatty liver disease and provided an additional contribution when applied with other anthropometric measures.

## Introduction

Fatty liver disease (FLD), defined as an excess accumulation of fat in hepatocytes, includes non-alcoholic fatty liver disease, alcoholic fatty liver disease, and steatosis related to other etiologies. In the US, more than 1/3 of adults have FLD [1]. It is estimated that 15% of adults have FLD in mainland China, of whom 90% appeared to have non-alcoholic FLD [2,3]. Non-alcoholic FLD, in particular, has become one of the major global public health challenges because it increases the risk of liver cirrhosis, metabolic syndrome (MetS), Type 2 diabetes mellitus [4] and cardiovascular diseases [5]. Early diagnosis and management would effectively improve its prognosis [6–9].

The “gold standard” method for FLD diagnosis is liver biopsy. Imaging methods and serum transaminases detection are also widely used. However, these methods require special instruments, and liver biopsy is invasive. In addition, the use of transaminases might result in missing a large number of non-alcoholic FLD cases owing to their low sensitivity. [10] Therefore, the use of transaminases is not encouraged by International guidelines [11,12]. Simpler and more feasible tools such as anthropometric indices are necessary for screening for FLD in large populations.

It is estimated that nearly 76% of obesity cases are accompanied by FLD [13]. A large number of studies have demonstrated that many simple anthropometric measurements such as body mass index (BMI), waist circumference (WC), waist-to-hip ratio (WHR) [14] and subcutaneous adipose tissue thickness [15] are good predictors of non-alcoholic FLD. Central obesity indices (e.g., WC, WHR) have performed better than general obesity measures (e.g., BMI) [14,16]. Cheung et al. [17] reported that of the anthropometric indices, dorsocervical lipohypertrophy was the measurement most strongly associated with the severity of steatohepatitis. Neck circumference (NC), a measurement of upper-body subcutaneous fat, has been proposed as a quicker, more reliable and easier-to-apply anthropometric marker of central obesity [18]. Several studies have illustrated that NC can predict MetS [19] and cardio-metabolic risk factors [20]. However, to the best of our knowledge, few studies have assessed the association between NC and FLD. A liver biopsy study found that NC was the only index among various obesity measures that differed between patients with and without steatosis among 283 HIV/HCV-co-infected patients [21]. Due to the limited evidence available, the NC-FLD association and the predictive value of NC for FLD have largely remained unclear. No study has addressed whether NC has additional value beyond the traditional anthropometric measurements in predicting FLD.

The aim of the present study was to examine the association between NC and FLD and to evaluate its predictive value for FLD in a large population of Chinese adults.

## Methods

### Study Participants

This cross-sectional study recruited a total of 4771 subjects who attended a regular health checkup at the Health Examination Center of the First Affiliated Hospital of Sun Yat-sen University (Guangzhou, South China) between May 2009 and April 2010. We excluded individuals with the following characteristics: a history of malignancy or thyroid diseases (n = 36), weight-controlling efforts (n = 48), current treatment for hypertension, diabetes or dyslipidemia (486), and a lack of liver ultrasound data (n = 148). Ultimately, 4053 subjects (2436 men and 1617 women between 20 and 88 years of age) were included in this study. The study was approved by the Ethics Committee of the First Affiliated Hospital of Sun Yat-sen University. Written informed consent was obtained from all participants.

### Measurements

**Clinical and anthropometric data**. Individual information was collected using medical history questionnaires, anthropometric indicators, biochemical indices from overnight fasting blood samples and abdominal ultrasound. Well-trained physicians obtained all anthropometric measurements from participants after they fasted for one night. The subjects stood upright, facing forward with their shoulders relaxed. Height and weight were measured with the subjects barefoot and wearing only undergarments using a portable stadiometer (Holtain, Crymmych, Wales) accurate to 0.1 cm and a digital scale (Hanson, Watford, Hertforshire, England) accurate to 0.1 kg. Neck circumference was measured as previously described [22], accurate to 0.5 mm. The top edge of a plastic tape was placed just below the laryngeal prominence and perpendicular to the longitudinal axis of the neck, with the head positioned in the Frankfort horizontal plane. WC was measured at the midpoint between the lowest rib and the iliac crest, within 1 mm. Hip circumference (HC) was measured at the widest point, within 1 mm. Body mass index (BMI) was calculated as the weight in kilograms divided by the square of the height in meters (kg/m^2^). WHR ratios were calculated by dividing WC by HC. Blood pressure was measured twice on the upper right arm, with the participant in a sitting position, after 15 minutes of rest, with a calibrated sphygmomanometer (Hawksley, WA Baum Co, USA), and the average measurement was recorded.

Overweight was defined as a BMI ≧24 kg/m^2^ for both genders [23]. Central obesity was defined as a WC≧80 cm in women and ≧90 cm in men or as a WHR≧0.85 in women and ≧0.90 in men [24,25]. Diabetes was defined as fasting plasma glucose (FPG) levels ≥7.0 mmol/L [26] or a self-reported history of diabetes confirmed by the doctor(s).

**Biochemical tests**. Blood samples were collected and analyzed in the biochemistry laboratory of the hospital. Serum total cholesterol and triglycerides were analyzed using enzymatic colorimetric tests. Serum high density lipoprotein cholesterol was measured using the elective inhibition method. A homogeneous enzymatic calorimetric test was used to measure low density lipoprotein cholesterol (Advia1650 Autoanalyzer, Byer Diagnostics Leverkusen Germany). Apolipoproteins A, B and E were measured by immunoturbidimetric assays. Serum alanine transaminase (ALT), aspartate transaminase (AST) and γ-glutamyl transpeptidase (GGT) were measured using an automated analyzer (Technicon Sequential Multiple Analyzer; Technicon Instruments Corporation, Tarrytown, NY).

**Abdominal ultrasound and diagnosis of FLD**. Real-time ultrasonography of the upper abdominal organs was performed for each participant by two experienced physicians using a scanner (X200, Toshiba Corporation, Japan). The physicians performing the ultrasonography were blinded to the clinical and laboratory results.

FLD was diagnosed and semi-quantitated according to Graif’s criteria [27], which was later adopted by the Chinese Society of Hepatology [28], based on abdominal ultrasonic findings combined with the patients’ medical histories, clinical symptoms and laboratory results. The ultrasonic diagnosis was defined as follows: 1) diffuse enhancement of the near-field echo in the hepatic region (stronger than in the kidney and spleen regions) and gradual attenuation of the far-field echo; 2) unclear display of intra-hepatic lacuna structures; 3) mild to moderate hepatomegaly with a round and blunt border; 4) color Doppler ultrasonography showing a reduction of the blood flow signal in the liver or a difficult-to-display signal with a normal distribution of blood flow; and 5) unclear or non-intact display of the envelope of the right liver lobe and diaphragm. A mild degree of fatty liver was diagnosed based on the first criterion and any one of items 2–4, a moderate degree of fatty liver was diagnosed based on the first criterion and any two of the subsequent items (2–4), and a severe degree of fatty liver was diagnosed based on the presence of items 1 and 5 and any two of items 2–4.

### Statistical analysis

Statistical analysis was carried out using SPSS version 19.0 (SPSS Inc., Chicago, IL, USA). All data were analyzed and reported by gender. We tested the normality of the continuous variables and normalized the skewed variables (ALT, AST and GGT) using logarithmic transformation. The normalized variables were used for subsequent analyses. Continuous data were reported as the mean ± standard deviation (SD) or the median/interquartile range, according to the normal distribution status. For categorical data, frequencies and percentages were reported. Mean differences between the FLD and non-FLD groups were tested using analysis of covariance after adjusting for age. Proportion differences were tested using the Wald chi-square test. Participants were classified into quartiles (Q1–Q4), with Q1 as the reference. Multivariate logistic regression analysis was used to calculate odds ratios (ORs) and 95% confidence intervals (CI) for FLD according to the NC quartiles after adjusting for age and BMI and WC and WHR, both independently and combined. Receiver operating characteristic curve analysis was employed to determine the area under the curves (AUCs) of NC in relation to FLD and its optimal sex-specific cut-off value. The additive interaction between NC and other anthropometric measurements was evaluated using logistic regression analysis, as suggested by Rothman et al. [29,30], with 3 indices: the relative excess risk due to interaction (RERI), the attributable proportion due to interaction (AP) and the synergy index with 95% CI. If there was no additive interaction, the 95% CI of RERI and AP included 0, whereas that of a synergistic index contained 1. All tests were 2-sided, and a *P*-value of <0.05 was considered statistically significant.

## Results

The study sample comprised 2436 men and 1617 women with a mean age of 45.3±12.1 years. Among the participants, 790 (32.4%) men and 263 (16.3%) women had FLD. The participants’ characteristics by FLD status are shown in Table 1. The average age of the participants with FLD (vs. non-FLD) was higher for women (*P*<0.001) but not for men. The participants with FLD also had higher NC values (*P*<0.001) and other anthropometric measures (body weight, WC, HC, BMI and WHR) (all *P*<0.001); greater values for blood pressure, fasting blood glucose, blood uric acid, total cholesterol, triglycerides, apolipoprotein B, apolipoprotein E and low density lipoprotein cholesterol, as well as ALT, AST and GGT (*P*<0.001), and lower high density lipoprotein cholesterol (all *P*<0.001) were obtained in both men and women compared with the participants without FLD. Consistently, a higher proportion of the FLD participants had diabetes, overweight and central obesity than in the non-FLD participants of both genders (all *P*<0.001).

**Table 1:** Characteristics of subjects with (FLD) and without (non-FLD) FLD (age-adjusted) (n = 4053).

	Women(n = 1617)					Men(n = 2436)				
Variables	FLD	(n = 263)	non-FLD	(n = 1354)		FLD	(n = 790)	non-FLD	(n = 1646)	
Continuous data	Mean	SD	Mean	SD	P^1^	Mean	SD	Mean	SD	P^1^
Age, years	51.0	10.0	43.5	11.3	<0.001	46.0	11.1	45.6	13.1	0.492
Height, cm	158.0	5.5	158.2	5.4	0.015	169.7	5.7	169.3	5.9	0.080
Weight, kg	63.16	9.06	55.42	7.37	<0.001	76.31	9.17	67.35	8.86	<0.001
Neck circumference, cm	34.15	2.40	32.13	2.05	<0.001	38.79	2.36	36.72	2.23	<0.001
Waist circumference, cm	84.29	8.52	74.72	8.11	<0.001	92.23	7.16	83.66	7.99	<0.001
Hip circumference, cm	96.80	6.42	92.08	5.59	<0.001	99.66	5.52	95.00	5.45	<0.001
Body mass index, kg/m^2^	25.29	3.24	22.15	2.78	<0.001	26.47	2.61	23.47	2.65	<0.001
Waist/hip ratio	0.87	0.06	0.81	0.06	<0.001	0.93	0.05	0.88	0.05	<0.001
Systolic blood pressure, mmHg	130.7	19.5	116.3	17.3	<0.001	129.6	16.1	122.8	15.9	<0.001
Diastolic blood pressure, mmHg	78.4	10.8	71.6	10.3	<0.001	82.0	11.7	76.4	11.0	<0.001
Triglycerides, mmol/L	1.79	1.19	1.14	0.83	<0.001	2.41	1.64	1.51	1.08	<0.001
Total cholesterol, mmol/L	5.90	1.15	5.43	1.05	<0.001	5.82	1.19	5.47	1.02	<0.001
High density lipoprotein, mmol/L	1.37	0.33	1.55	0.38	<0.001	1.15	0.28	1.29	0.38	<0.001
Low density lipoprotein, mmol/L	3.65	0.98	3.27	0.92	0.004	3.61	0.99	3.46	0.95	<0.001
Fasting blood glucose, mmol/L	5.32	1.22	4.81	0.62	<0.001	5.36	1.47	4.94	1.02	<0.001
Blood uric acid, μmol/L	272.2	79.4	215.0	64.2	<0.001	359.0	85.7	314.0	74.8	<0.001
Apolipoprotein A, g/L	1.31	0.33	1.39	0.40	0.002	1.17	0.36	1.20	0.29	0.073
Apolipoprotein B, g/L	0.99	0.23	0.87	0.23	<0.001	1.00	0.24	0.93	0.30	<0.001
Apolipoprotein E, g/L	47.26	14.66	42.28	12.38	<0.001	48.87	16.40	40.69	12.64	<0.001
Aspartate transaminase^3^, U/L	22.0	(20.0–27.0)	20.0	(17.0–22.0)	<0.001	26.0	(22.0–33.0)	22.0	(22.0–26.0)	<0.001
Alanine transaminase^3^, U/L	21.0	(15.0–31.0)	15.0	(11.0–19.0)	<0.001	34.0	(23.0–48.0)	21.0	(16.0–28.0)	<0.001
γ-glutamyl transpeptidase^3^, U/L	24.0	(18.0–34.5)	17.0	(13.0–22.0)	<0.001	44.0	(30.8–65.0)	27.0	(20.0–39.0)	<0.001
Categorical data	n	%	n	%	P^2^	n	%	n	%	P^2^
Diabetes^4^	20	7.60	27	1.99	<0.001	83	10.51	59	3.58	<0.001
Overweight^5^	172	65.40	301	22.23	<0.001	668	84.56	695	42.22	<0.001
Central obesity^6^	190	72.24	331	24.45	<0.001	480	60.76	369	22.42	<0.001

*P* ^1^: Covariance analysis adjusted for age

*P* ^2^: Wald chi-square test adjusted for age

^3^: Median (percentile 25, 75). Logarithmic transformation before analysis

^4^: Diabetes was defined as fasting plasma glucose (FPG) levels ≥7.0 mmol/L or by a self-reported history of diabetes confirmed by the doctor(s).

^5^: Overweight was defined as a BMI ≧24 kg/m^2^ for both genders.

^6^: Central obesity was defined as a waist circumference (WC)≧80 cm in women and ≧90 cm in men.

Logistic regression analysis showed a strong positive association between NC and FLD in both men and women after adjusting for age (both *P-*trends <0.001). The OR (95% CI) of FLD for Q4 (vs. Q1) of NC was 7.70 (4.95–11.99) for women and 12.42 (9.22–16.74) for men. After further adjusting for BMI and WC, both independently and combined, the corresponding ORs were attenuated to 1.94–2.53 (all *P-*trends<0.001) in women and 1.45–2.08 (all *P*-trends<0.05) in men. The NC-FLD association remained significant even when the analysis was adjusted for BMI, WC and WHR. The OR (95% CI) was 2.09 (1.20–3.63) for women and 1.65 (1.11–2.45) for men (Table 2).

**Table 2:** Odds ratios of FLD by quartiles of neck circumference.

	FLD n (%)	Model 1 (Age-adjusted)	Model 2 (Model 1+BMI)	Model 3 (Model 1+WC)	Model 4 (Model 1+ BMI+WC)	Model 5 (Model 1+BMI+WC+WHR)
Women						
Q1 (27.0–31.0)	28 (5.7)	1.00	1.00	1.00	1.00	1.00
Q2 (31.1–32.2)	32 (9.8)	1.67 (0.98–2.85)	1.10 (0.64–1.92)	1.08 (0.62–1.88)	1.01 (0.58–1.76)	1.03 (0.59–1.79)
Q3 (32.3–33.8)	63 (15.0)	2.58 (1.60–4.13)**	1.32 (0.79–2.19)	1.25 (0.75–2.09)	1.13 (0.67–1.90)	1.17 (0.70–1.96)
Q4 (33.9–41.5)	140 (36.7)	7.70 (4.95–11.99) **	2.53 (1.50–4.27) **	2.29 (1.34–3.93)*	1.94 (1.12–3.36) *	2.09 (1.20–3.63) *
*P*-trend		<0.001	<0.001	<0.001	0.007	0.003
Men						
Q1 (28.5–35.8)	72 (11.4)	1.00	1.00	1.00	1.00	1.00
Q2 (35.9–37.3)	133 (22.3)	2.23 (1.63–3.05) **	1.06 (0.76–1.48)	1.12 (0.80–1.57)	0.95 (0.68–1.34)	0.99 (0. 70–1.40)
Q3 (37.4–39.0)	237 (37.0)	4.58 (3.42–6.14) **	1.49 (1.07–2.07) *	1.48 (1.06–2.06) *	1.19 (0.84–1.67)	1.29 (0.92–1.83)
Q4 (39.1–50.1)	348 (61.5)	12.42 (9.22–16.74) **	2.04 (1.40–2.97) **	2.08 (1.43–3.03) **	1.45 (0.98–2.15)	1.65 (1.11–2.45) *
*P*-trend		<0.001	<0.001	<0.001	0.015	0.002

Model 1: adjusted for age;

Model 2: adjusted for age and BMI;

Model 3: adjusted for age and WC;

Model 4: adjusted for age, BMI and WC;

Model 5: adjusted for age, BMI, WC and WHR.

*: p<0.05 compared with Q1;

**: p<0.001 compared with Q1.

The receiver operating characteristic curve analysis of NC for FLD was performed. The AUCs (95% CI) were 0.744 (0.711–0.777) and 0.744 (0.723–0.764) in women and men, respectively. An NC cutoff point of 34 cm in women and 38 cm in men had the optimal sensitivity and specificity to predict subjects with FLD. The sensitivity and specificity were 0.62 and 0.77 in women and 0.66 and 0.71 in men, respectively.

**Fig 1:**
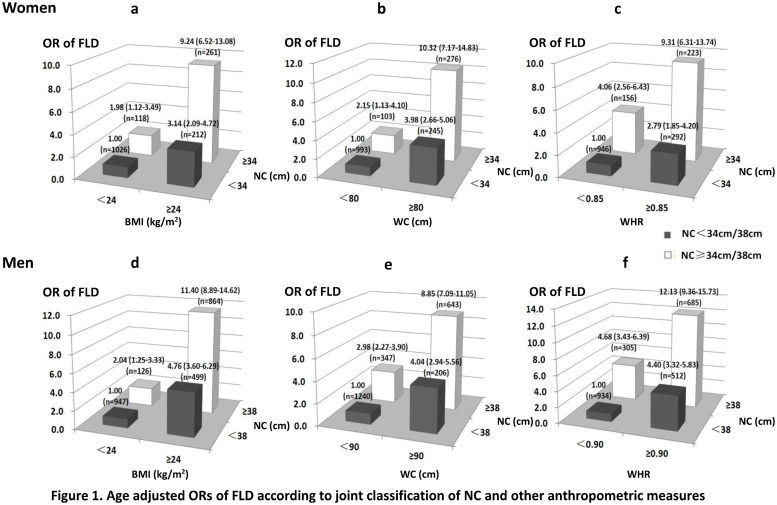
Age-adjusted ORs of FLD according to joint classification of NC and other anthropometric measures.

We further examined the combined effects between NC (<34/38 vs. ≥34/38 cm, women/men) and BMI (<24 vs. ≥24 kg/m^2^), WC (<80/90 vs. ≥80/90 cm, women/men) or WHR (<0.85/0.90 vs. ≥0.85/0.90, women/men) in predicting FLD using a stratified analysis (Fig. 1). In the participants with high NC values, the ORs (95% CI) of FLD for the group with high BMI, WC or WHR were 9.24 (6.52–13.08), 10.32 (7.17–14.83) and 9.31 (6.31–13.74) in women and 11.40 (8.89–14.62), 8.85 (7.09–11.05) and 12.13 (9.36–15.73) in men, respectively, which were much greater than those of 2.79–3.98 (women) and 4.04–4.76 (men) in the participants with low NC values. A high NC value was associated with a significantly greater risk of FLD, even in participants with a normal BMI, WC or WHR. The additive interaction of NC and other anthropometric measures on FLD was evaluated. The RERI (95% CI) of NC vs. BMI, WC or WHR was 5.12 (2.27–7.97), 5.19 (2.00–8.38), and 3.46 (0.38–6.55) in women and 5.60 (3.36–7.83), 2.83 (0.84–4.81), and 4.05 (1.63–6.47) in men, respectively. The AP values (95% CI) were 0.55 (0.36–0.75), 0.50 (0.30–0.71), and 0.37 (0.12–0.62) in women and 0.49 (0.36–0.62), 0.32 (0.13–0.51), and 0.33 (0.18–0.49) in men. The synergy indexes (95% CI) were 2.64 (1.52–4.59), 2.26 (1.37–3.71), and 1.72 (1.08–2.73) in women and 2.17 (1.59–2.96), 1.56 (1.13–2.16), and 1.57 (1.21–2.03) in men, respectively. These indicated an additive interaction between NC and other anthropometric measures.

## Discussion

In this large cross-sectional study of urban residents in southern China, we investigated the association between NC and FLD and its predictive value for FLD. NC was significantly associated with the prevalence of FLD, as diagnosed by abdominal ultrasound, and had an independent predictive value after adjusting for age, BMI, WC and WHR. NC had synergistic value in predicting FLD when combined with BMI, WC and WHR.

In the present study, we confirmed the independent contribution of NC for FLD beyond other anthropometric measures (BMI, WC and WHR) in women and men. The close associations between the traditional anthropometric measures of BMI, WC and WHR and MetS, cardiovascular diseases [31] and fatty liver disease [32] are well established. Several recent studies have shown that neck circumference was a simple and feasible anthropometric marker of upper-body fat deposits [33]. Many other studies have also found independent associations between NC and risk factors of metabolic syndrome and cardiovascular disease. A cross-sectional study from Turkey that enrolled 1912 middle-aged and elderly adults demonstrated that NC had a greater value than BMI and WC regarding the association with MetS and its components [18]. The results of the Framingham Heart Study [34] indicated that NC was independently associated with cardiovascular disease risk factors beyond visceral adipose tissue and BMI. The same result of a positive association between NC and MetS risk factors was also observed in the Brazilian Metabolic Syndrome Study [19]. In this study, we found that the risk of having FLD in the highest (vs. the lowest) quartile was increased by 109% (95% CI: 20%–263%) in women and 65% (95% CI: 11%–145%) in men after adjusting for age, BMI, WC and WHR. To the best of our knowledge, this is the first study to demonstrate the independent NC-FLD association and its additional predictive value in a general population.

Previous research has tried to assess the combined impact of two or more physical indicators on the risk of adiposity-related disease but generated inconsistent findings. Iwao et al. [35] observed that WC only modestly added to BMI in the prediction of cardiovascular disease risk factors in younger subjects and added little predictive value in older subjects (n = 1941). A prospective study of 4107 men aged 60–79 [36] suggested that the combination of WC and mid-arm muscle circumference provided the best estimation of the total mortality risk in the elderly. Importantly, a significant synergistic effect between NC and visceral adipose tissue on cardio-metabolic risk factors was noted in the Framingham Heart Study [37]. However, waist indices, including WC, the waist-to-height ratio or WHR, did not enhance the prediction of hypertension on the basis of BMI in a cross-sectional study of 7,336 Chinese adults [38]. Consistent with previous findings, we first reported a significant synergistic effect between NC and BMI, WC or WHR on FLD. The findings of our study and previous studies [37] suggest that the combination of NC and other obesity indices would be helpful to better predict FLD and, possibly, other cardiovascular diseases risk factors.

**Fig 2:**
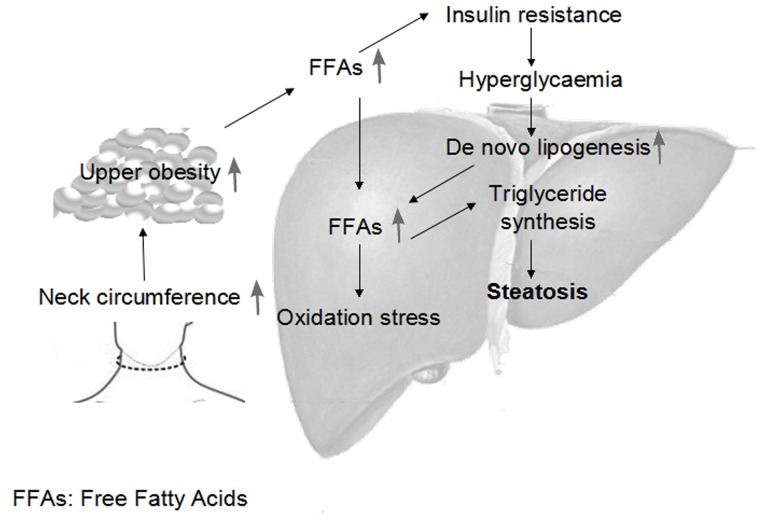
Mechanisms linking a hypertrophic neck with the development of fatty liver disease.

NC was independently correlated with FLD in this study and with cardiometabolic risk factors beyond other adiposity measures in previous reports [20,37]. The potential mechanism might be related to upper-body fat [39,40], which could be estimated by the NC [34,37]. Upper-body obesity causes metabolic abnormalities, including increased circulating free fatty acids (FFAs) [41]. The excess FFAs may contribute to the development of FLD by (1) contributing to triglyceride formation and storage in the liver, as Donnelly et al. [42] reported that 59% of hepatic fat is derived from circulating FFAs, with lesser contributions from de novo lipogenesis (26%) and diet (15%), (2) inducing insulin resistance, which is thought to be related to the first “hit” in the multistep pathogenesis of non-alcoholic fatty liver disease, or (3) increasing oxidative stress, thereby triggering the inflammatory response and progressive liver damage. Stojiljkovic et al. [43] reported that an acute increase in plasma lipids increased the concentration of the oxidative stress biomarker F2-isoprostanes and raised the possibility of the cardiovascular risk factor cluster. These observations might explain the mechanism by which an increased neck circumference independently increases the risk of developing fatty liver disease. (Fig. 2)

We identified optimal NC cutoffs of ≥34 cm in women and ≥38 cm in men for the prediction of FLD via receiver operating characteristic curve analysis. The cutoffs were similar to those for MetS classification (women: 33 cm; men: 37 cm) reported in our previous article [20]. FLD and MetS share a common pathogenesis, including adverse metabolic profiles such as insulin resistance, dyslipidemia and systematic inflammation, and many studies have explored their correlation. Our research added evidence to support FLD as the hepatic manifestation of MetS. Consistent with our findings, similar NC cutoffs were obtained for the prediction of diabetes in elderly residents in Eastern China (women: 35 cm; men: 38 cm) [44], as well as in a Turkish population (women: 35 cm; men: 39 cm), for the prediction of MetS [18]. These results suggested NC cutoffs for the prediction of FLD and cardiovascular disease risk factors. Further longitudinal studies are needed to validate the NC cutoffs.

One of the limitations of our study is the cross-sectional design, which might limit the causal inference. Next, our results might not be fully applicable to other racial or ethnic groups or populations in which the prevalence of FLD is much lower. In addition, our study sample was from a population receiving health check-ups. There was limited information for us to perform additional stratified analysis to adjust for the influence of alcoholic consumption, viral hepatitis, smoking status, the degree of physical activity and fasting insulin levels. We could not exclude the potential confounding bias caused by these factors. However, alcoholic fatty liver disease is unlikely to affect the NC-FLD association substantially considering that 1) non-alcoholic fatty liver disease currently accounts for the majority of FLD in China [2], 2) there is a correlation between alcoholic fatty liver disease and adiposity because alcoholics tend to have a high-calorie diet pattern, which may lead to a higher risk of obesity [45], and 3) the indistinguishable spectrum of the histological features of both alcoholic fatty liver disease and non-alcoholic fatty liver disease suggests a possible convergence of pathogenic mechanisms between the two conditions [46]. Therefore, we believed that it was valuable to examine the association between NC and FLD without distinguishing between alcoholic and non-alcoholic fatty liver disease. Finally, we could not compare the validity of NC with that of abdominal visceral adipose tissue due to the lack of a direct and more accurate measure.

In conclusion, NC was greater in FLD subjects than in non-FLD subjects and was independently associated with the prevalence of FLD. NC is a valuable predictor of FLD, especially when applied with other anthropometric measures

## Supporting Information

S1 Dataset Research Dataset. (XLS)

## References

[1] Angulo P (2007) GI epidemiology: nonalcoholic fatty liver disease. Alimentary pharmacology & therapeutics 25: 883–889. 10.1155/2014/953181 17402991

[2] Zhou YJ, Li YY, Nie YQ, Ma JX, Lu LG, et al (2007) Prevalence of fatty liver disease and its risk factors in the population of South China. World J Gastroenterol 13: 6419–6424. 18081233 10.3748/wjg.v13.i47.6419 PMC4205463

[3] Fan JG, Zhu J, Li XJ, Chen L, Li L, et al (2005) Prevalence of and risk factors for fatty liver in a general population of Shanghai, China. J Hepatol 43: 508–514. 16006003 10.1016/j.jhep.2005.02.042

[4] Targher G, Chonchol M, Bertolini L, Rodella S, Zenari L, et al (2008) Increased risk of CKD among type 2 diabetics with nonalcoholic fatty liver disease. Journal of the American Society of Nephrology 19: 1564–1570. 10.1681/ASN.2007101155 18385424 PMC2488256

[5] Targher G, Arcaro G (2007) Non-alcoholic fatty liver disease and increased risk of cardiovascular disease. Atherosclerosis 191: 235–240. 16970951 10.1016/j.atherosclerosis.2006.08.021

[6] Machado MV, Cortez-Pinto H (2014) Management of fatty liver disease with the metabolic syndrome. Expert review of gastroenterology & hepatology: 1–14. 10.1586/17474124.1.1.1 24665862

[7] Athyros VG, Tziomalos K, Gossios TD, Griva T, Anagnostis P, et al (2010) Safety and efficacy of long-term statin treatment for cardiovascular events in patients with coronary heart disease and abnormal liver tests in the Greek Atorvastatin and Coronary Heart Disease Evaluation (GREACE) Study: a post-hoc analysis. Lancet 376: 1916–1922. 10.1016/S0140-6736(10)61272-X 21109302

[8] Vilar Gomez E, Rodriguez De Miranda A, Gra Oramas B, Arus Soler E, Llanio Navarro R, et al (2009) Clinical trial: a nutritional supplement Viusid, in combination with diet and exercise, in patients with nonalcoholic fatty liver disease. Aliment Pharmacol Ther 30: 999–1009. 10.1111/j.1365-2036.2009.04122.x 19691668

[9] Satapathy SK, Sakhuja P, Malhotra V, Sharma BC, Sarin SK (2007) Beneficial effects of pentoxifylline on hepatic steatosis, fibrosis and necroinflammation in patients with non-alcoholic steatohepatitis. J Gastroenterol Hepatol 22: 634–638. 17444848 10.1111/j.1440-1746.2006.04756.x

[10] Mofrad P, Contos MJ, Haque M, Sargeant C, Fisher RA, et al (2003) Clinical and histologic spectrum of nonalcoholic fatty liver disease associated with normal ALT values. Hepatology 37: 1286–1292. 12774006 10.1053/jhep.2003.50229

[11] Prati D, Taioli E, Zanella A, Della Torre E, Butelli S, et al (2002) Updated definitions of healthy ranges for serum alanine aminotransferase levels. Annals of internal medicine 137: 1–10. 12093239 10.7326/0003-4819-137-1-200207020-00006

[12] Nascimbeni F, Pais R, Bellentani S, Day CP, Ratziu V, et al (2013) From NAFLD in clinical practice to answers from guidelines. J Hepatol 59: 859–871. 10.1016/j.jhep.2013.05.044 23751754

[13] Adams LA, Angulo P, Lindor KD (2005) Nonalcoholic fatty liver disease. Canadian Medical Association Journal 172: 899–905. 15795412 10.1503/cmaj.045232 PMC554876

[14] Zheng R-D, Chen Z-R, Chen J-N, Lu Y-H, Chen J (2012) Role of body mass index, waist-to-height and waist-to-hip ratio in prediction of nonalcoholic fatty liver disease. Gastroenterology research and practice 2012 10.1155/2012/897678 22701476 PMC3369513

[15] Ayonrinde OT, Olynyk JK, Beilin LJ, Mori TA, Pennell CE, et al (2011) Gender-specific differences in adipose distribution and adipocytokines influence adolescent nonalcoholic fatty liver disease. Hepatology 53: 800–809. 10.1002/hep.24097 21374659

[16] Meng L, Luo N, Mi J (2011) Impacts of types and degree of obesity on non-alcoholic fatty liver disease and related dyslipidemia in Chinese school-age children? Biomed Environ Sci 24: 22–30. 10.3967/0895-3988.2011.01.003 21440836

[17] Cheung O, Kapoor A, Puri P, Sistrun S, Luketic VA, et al (2007) The impact of fat distribution on the severity of nonalcoholic fatty liver disease and metabolic syndrome. Hepatology 46: 1091–1100. 17610277 10.1002/hep.21803

[18] Onat A, Hergenç G, Yüksel H, Can G, Ayhan E, et al (2009) Neck circumference as a measure of central obesity: associations with metabolic syndrome and obstructive sleep apnea syndrome beyond waist circumference. Clinical nutrition 28: 46–51. 10.1016/j.clnu.2008.10.006 19010573

[19] Stabe C, Vasques ACJ, Lima MMO, Tambascia MA, Pareja JC, et al (2013) Neck circumference as a simple tool for identifying the metabolic syndrome and insulin resistance: results from the Brazilian Metabolic Syndrome Study. Clinical endocrinology 78: 874–881. 10.1111/j.1365-2265.2012.04487.x 22804918

[20] Zhou JY, Ge H, Zhu MF, Wang LJ, Chen L, et al (2013) Neck circumference as an independent predictive contributor to cardio-metabolic syndrome. Cardiovasc Diabetol 12: 76 10.1186/1475-2840-12-76 23680280 PMC3661343

[21] Rodriguez-Torres M, Govindarajan S, Sola R, Clumeck N, Lissen E, et al (2008) Hepatic steatosis in HIV/HCV co-infected patients: correlates, efficacy and outcomes of anti-HCV therapy: a paired liver biopsy study. J Hepatol 48: 756–764. 10.1016/j.jhep.2008.01.015 18314217

[22] Ben-Noun L, Sohar E, Laor A (2001) Neck circumference as a simple screening measure for identifying overweight and obese patients. Obes Res 9: 470–477. 11500527 10.1038/oby.2001.61

[23] Zhou BF, Cooperative Meta-Analysis Group of the Working Group on Obesity in C (2002) Predictive values of body mass index and waist circumference for risk factors of certain related diseases in Chinese adults—study on optimal cut-off points of body mass index and waist circumference in Chinese adults. Biomed Environ Sci 15: 83–96. 12046553

[24] Alberti KG, Zimmet P, Shaw J (2006) Metabolic syndrome—a new world-wide definition. A Consensus Statement from the International Diabetes Federation. Diabet Med 23: 469–480. 16681555 10.1111/j.1464-5491.2006.01858.x

[25] Yusuf S, Hawken S, Ounpuu S, Dans T, Avezum A, et al (2004) Effect of potentially modifiable risk factors associated with myocardial infarction in 52 countries (the INTERHEART study): case-control study. Lancet 364: 937–952. 15364185 10.1016/S0140-6736(04)17018-9

[26] Association AD (2012) Diagnosis and classification of diabetes mellitus. Diabetes Care 35 Suppl 1: S64–71. 10.2337/dc12-s064 22187472 PMC3632174

[27] Graif M, Yanuka M, Baraz M, Blank A, Moshkovitz M, et al (2000) Quantitative estimation of attenuation in ultrasound video images: correlation with histology in diffuse liver disease. Invest Radiol 35: 319–324. 10803673 10.1097/00004424-200005000-00006

[28] Zeng MD, Fan JG, Lu LG, Li YM, Chen CW, et al (2008) Guidelines for the diagnosis and treatment of nonalcoholic fatty liver diseases. J Dig Dis 9: 108–112. 10.1111/j.1751-2980.2008.00331.x 18419645

[29] Rothman KJ, Greenland S, Walker AM (1980) Concepts of interaction. Am J Epidemiol 112: 467–470. 7424895 10.1093/oxfordjournals.aje.a113015

[30] Andersson T, Alfredsson L, Kallberg H, Zdravkovic S, Ahlbom A (2005) Calculating measures of biological interaction. Eur J Epidemiol 20: 575–579. 16119429 10.1007/s10654-005-7835-x

[31] Wing RR, Jeffery RW, Burton LR, Thorson C, Kuller LH, et al (1992) Change in waist-hip ratio with weight loss and its association with change in cardiovascular risk factors. Am J Clin Nutr 55: 1086–1092. 1595579 10.1093/ajcn/55.6.1086

[32] Gray B, Muhlhausler BS, Davies PS, Vitetta L (2013) Liver enzymes but not free fatty acid levels predict markers of insulin sensitivity in overweight and obese, nondiabetic adults. Nutr Res 33: 781–788. 10.1016/j.nutres.2013.07.019 24074735

[33] Aswathappa J, Garg S, Kutty K, Shankar V (2013) Neck circumference as an anthropometric measure of obesity in diabetics. N Am J Med Sci 5: 28–31. 10.4103/1947-2714.106188 23378952 PMC3560135

[34] Preis SR, Pencina MJ, D’Agostino RB Sr, Meigs JB, Vasan RS, et al (2013) Neck circumference and the development of cardiovascular disease risk factors in the Framingham Heart Study. Diabetes Care 36: e3 10.2337/dc12-0738 23264305 PMC3526209

[35] Iwao S, Iwao N, Muller DC, Elahi D, Shimokata H, et al (2001) Does waist circumference add to the predictive power of the body mass index for coronary risk? Obes Res 9: 685–695. 11707535 10.1038/oby.2001.93

[36] Wannamethee SG, Shaper AG, Lennon L, Whincup PH (2007) Decreased muscle mass and increased central adiposity are independently related to mortality in older men. Am J Clin Nutr 86: 1339–1346. 17991644 10.1093/ajcn/86.5.1339

[37] Preis SR, Massaro JM, Hoffmann U, D’Agostino RB Sr, Levy D, et al (2010) Neck circumference as a novel measure of cardiometabolic risk: the Framingham Heart study. J Clin Endocrinol Metab 95: 3701–3710. 10.1210/jc.2009-1779 20484490 PMC2913042

[38] Tuan NT, Adair LS, Stevens J, Popkin BM (2010) Prediction of hypertension by different anthropometric indices in adults: the change in estimate approach. Public Health Nutr 13: 639–646. 10.1017/S1368980009991479 19758482 PMC2855402

[39] Ballestri S, Lonardo A, Carulli L, Ricchi M, Bertozzi L, et al (2008) The neck-liver axis. Madelung disease as further evidence for an impact of body fat distribution on hepatic histology. Hepatology 2008 Jan;47(1):361–2. 18161705 10.1002/hep.21977

[40] Lonardo A, Caldwell SH, Loria P (2010) Clinical physiology of NAFLD: a critical overview of pathogenesis and treatment. Expert Review of Endocrinology & Metabolism 5: 403–423. 10.14423/SMJ.0000000000000219 25580750

[41] Nielsen S, Guo Z, Johnson CM, Hensrud DD, Jensen MD (2004) Splanchnic lipolysis in human obesity. Journal of Clinical Investigation 113: 1582–1588. 15173884 10.1172/JCI21047 PMC419492

[42] Donnelly KL, Smith CI, Schwarzenberg SJ, Jessurun J, Boldt MD, et al (2005) Sources of fatty acids stored in liver and secreted via lipoproteins in patients with nonalcoholic fatty liver disease. Journal of Clinical Investigation 115: 1343–1351. 15864352 10.1172/JCI23621 PMC1087172

[43] Stojiljkovic MP, Lopes HF, Zhang D, Morrow JD, Goodfriend TL, et al (2002) Increasing plasma fatty acids elevates F2-isoprostanes in humans: implications for the cardiovascular risk factor cluster. Journal of hypertension 20: 1215–1221. 12023694 10.1097/00004872-200206000-00036

[44] Yan Q, Sun D, Li X, Zheng Q, Li L, et al (2014) Neck circumference is a valuable tool for identifying metabolic syndrome and obesity in Chinese elder subjects: a community-based study. Diabetes Metab Res Rev 30: 69–76. 10.1002/dmrr.2464 23996612

[45] Chakraborty S (2014) Analysis of NHANES 1999–2002 data reveals noteworthy association of alcohol consumption with obesity. Ann Gastroenterol 27: 250–257. 24974978 PMC4073022

[46] Volzke H (2012) Multicausality in fatty liver disease: is there a rationale to distinguish between alcoholic and non-alcoholic origin? World J Gastroenterol 18: 3492–3501. 10.3748/wjg.v18.i27.3492 22826613 PMC3400850

